# Metacognitive Therapy for Emotional Distress in Adult Cancer Survivors: A Case Series

**DOI:** 10.1007/s10608-017-9862-9

**Published:** 2017-05-29

**Authors:** Peter L. Fisher, Angela Byrne, Peter Salmon

**Affiliations:** 1 Psychological Sciences, University of Liverpool, Whelan Buliding, Liverpool, L69 3GB UK; 2 Liverpool Psychology Cancer Service, Royal Liverpool and Broadgreen NHS Trust, Liverpool, UK; 3 Nidaros, Østmarka University Hospital, Trondheim, Norway

**Keywords:** Cancer survivors, Emotional distress, Metacognitive therapy, Case series study

## Abstract

Many adult cancer survivors experience persistent emotional distress after completing cancer treatment. The aim of this study was to test the potential of a brief transdiagnostic psychological intervention—metacognitive therapy (MCT)—in reducing emotional distress in adult cancer survivors. A non-concurrent multiple baseline design with 3- and 6-months follow-up was used to evaluate the effects of MCT in four patients consecutively referred to a psycho-oncology service. Each patient received six 1-h sessions of MCT. Anxiety, depression, worry/rumination, fear of cancer recurrence and metacognitive beliefs were assessed using self-report questionnaires. MCT was associated with clinically significant reductions in anxiety, depression, fear of cancer recurrence, worry/rumination and metacognitive beliefs at the end of treatment, and gains were maintained in all patients to 3-months follow-up and in three out of four patients to 6-months follow-up. MCT is a promising brief transdiagnostic approach to psychological morbidity in adult survivors of cancer. Larger scale controlled trials are now required.

## Introduction

Cancer is becoming a long-term health condition, with approximately 70% of patients living for at least 5 years from the time of diagnosis (Cancer Research UK). Psychological distress commonly occurs in cancer survivors. A systematic review on the prevalence of anxiety and depression in cancer survivors at least 2 years post-diagnosis reported prevalence of 11.6% for depression and 17.9% for anxiety (Mitchell et al. 2013). An earlier review concluded that approximately 25% of adult cancer survivors experience levels of anxiety and depression warranting treatment (Hoffman et al. 2009). Fear of cancer recurrence (FCR), defined as worry about cancer returning or advancing in the same or a different body part (Vickberg 2003), is also very common in cancer survivors. Across tumour groups, approximately 56% of survivors experience moderate to high FCR (Simard et al. 2013). Psychiatric comorbidity is common in cancer survivors; anxiety and depression often co-occur (Mehnert and Koch 2008) and, in survivors with clinical levels of FCR, psychiatric comorbidity is the rule rather than exception (Simard and Savard 2015). Anxiety, depression and FCR are all linked to poorer quality of life (Lebel et al. 2013) and increased healthcare use and costs (Sarkar et al. 2015).

The development of psychological interventions for cancer patients has tended to follow the disorder-specific approach used in mental health. Disorder-specific approaches focus on one clinical problem at a time; therefore, distinct protocols exist for treating anxiety and depression in cancer patients and survivors (e.g. Greer et al. 2010; Hopko et al. 2007). More recently, protocols have been developed to address FCR also (e.g. Butow et al. 2013; Maheu et al. 2016). However, a disorder-specific approach to psychological morbidity in cancer survivors may not be the most cost-effective or efficacious approach Disorder-specific approaches require interventions to be tailored to the main presenting complaint, which could limit their clinical utility because of the comorbidity of anxiety, depression and FCR in cancer survivors. Disorder-specific approaches may also limit the dissemination of psychological treatments as they require healthcare professionals to be trained in different therapeutic models and associated interventions (Norton and Paulus 2016). A transdiagnostic approach offers a more cost-effective model of training, requiring practitioners to develop competency in a single treatment protocol applicable to patients regardless of their specific symptoms of distress (McEvoy et al. 2009). Furthermore, transdiagnostic approaches may offer patients a more time-efficient intervention because comorbid problems could be treated concurrently, rather than sequentially by disorder specific interventions. Basing intervention on a transdiagnostic model of psychopathology which can account for all forms of psychological distress would be a significant advance in treating cancer survivors.

Metacognitive therapy is based on the transdiagnostic model of metacognitive processes in psychopathology, the Self-regulatory executive function (S-REF) model (Wells and Matthews 1994). The S-REF model states that all forms of emotional disorder are maintained by the cognitive-attentional syndrome (CAS), which comprises three processes: (i) perseverative thinking (e.g. worry, rumination, over-analysing); (ii) inflexible self-focused attention (monitoring for signs of threat); and (iii) counterproductive coping strategies that impair cognitive and emotional regulation. Cancer survivors experience many types of negative thoughts (e.g. thoughts about cancer returning, memories or images of their cancer treatment, thoughts of loss), which can result in further conceptual processing, such as worrying about coping with cancer recurrence or ruminating about implications of cancer on work and family roles. For most patients, worry and rumination are transient but, in those who will become depressed or anxious, they persist.

The S-REF model specifies that the CAS is activated and guided by positive and negative metacognitive beliefs. Positive metacognitive beliefs concern the usefulness of worry, ruminating, threat monitoring and coping strategies e.g. “worrying will help me cope”, “monitoring my symptoms constantly keeps me safe”. Unfortunately, worry/rumination and each aspect of the CAS are counter-productive, because they increase negative thoughts and broaden the sense of threat. The individual responds as if the negative thought is valid and important, preventing the development of a more flexible relationship with negative thoughts that can reduce worry/rumination. Similarly, the S-REF model explains how threat monitoring (e.g. scanning for symptoms or for negative thoughts) and maladaptive coping behaviours (e.g. avoiding reminders of cancer, withdrawal, distraction) are driven by metacognitive beliefs that these strategies will be helpful. However, the coping strategies have the opposite effect by maintaining the sense of threat and personal vulnerability so that emotional distress persists or escalates. Within the S-REF model, negative metacognitive beliefs are of fundamental importance to persistent emotional distress. There are two main domains of negative metacognitive beliefs: that perseverative thinking is uncontrollable (e.g. “I cannot control my worry”, “I can’t stop analysing my past mistakes”) and that it can be harmful mentally and/or physically (e.g. “I could lose control of my mind”). Activation of these negative metacognitive beliefs leads to worry about worry, which in turn increases distress. Furthermore, negative metacognitive beliefs concerning the uncontrollability of perseverative thinking result in limited effort to stop worry/rumination as the individual believes it is not possible to do so, thereby further maintaining distress.

Considerable evidence supports the association between metacognitive beliefs and emotional distress in a range of anxiety and depressive disorders in mental health (e.g. Wells 2013), with emerging evidence in several physical health populations including chronic fatigue (Maher-Edwards et al. 2011), epilepsy (Fisher et al. 2016), and Parkinson’s disease (Brown and Fernie 2015). Recent studies suggest that metacognitive theory and therapy can be translated to cancer patients and survivors specifically. In patients with recently diagnosed breast or prostate cancer, metacognitive beliefs were associated with anxiety, depression and trauma symptoms after controlling for negative health beliefs (Cook et al. 2015a). In a prospective study, breast and prostate cancer patients’ metacognitive beliefs around the time of diagnosis predicted anxiety, depression and trauma symptoms 12 months later, after controlling for baseline symptoms and metacognitive beliefs (Cook et al. 2015b). Metacognitive beliefs are also significantly higher in breast cancer patients with clinical levels of FCR compared to those patients without FCR (Butow et al. 2015).

Key components of the CAS have also been linked to heightened emotional distress in cancer survivors. Worry about general health and cancer is associated with elevated anxiety and depression (Deimling et al. 2006). The association between worry about cancer recurrence or progression and distress has been extensively documented (Simard et al. 2013). In cancer survivors, rumination in response to negative thoughts is associated with greater distress (Morris and Shakespeare-Finch 2011) and mediates the relationship between harm/loss cognitions and depression (Steiner et al. 2014). There is also emerging evidence that anxious cancer patients show an attentional bias for threat-related stimuli (e.g. Chan et al. 2011; Butow et al. 2015) which is consistent with the role of maladaptive attentional processing in the S-REF model.

There has been one treatment study of MCT in cancer patients: an open trial in young adult survivors of paediatric cancer (Fisher et al. 2015). MCT reduced anxiety, trauma symptoms and depression, with treatment gains maintained through to 6-months follow-up. In that study, MCT was delivered according to a transdiagnostic model (Wells 2009) over an average of nine 1-h sessions the intervention is described in a case study (McNicol et al. 2013). Reducing the costs of psychological interventions in cancer care by providing brief interventions is important given the limited resources of public healthcare systems (Jansen et al. 2016). MCT has been successfully delivered over only 6–8 sessions for depression (Wells et al. 2012) post-traumatic stress disorder (Wells et al. 2015) and health anxiety (Bailey and Wells 2014), but such brief forms of MCT have not yet been tested in a cancer population. Therefore, the aim of this case series is to evaluate whether MCT delivered in six 1-h session**s** could potentially reduce emotional distress, FCR and metacognitive beliefs and processes in adult cancer survivors.

## Method

### Design

A non-concurrent multiple baseline design (Watson and Workman 1981) with 3- and 6-month follow-up was used to evaluate the effectiveness of MCT in reducing distress in cancer survivors. Patients were randomly assigned to no-treatment baselines of 3–6 weeks. Each patient was to start treatment at the predefined time, provided the baseline was stable, defined as limited variability in the range of scores and an absence of a clear increasing or decreasing trend. In practice, patients’ commitments delayed the start of treatment. Therefore, two patients began treatment after a 5-week baseline and two began after a 6-week baseline.

### Participants

The participants were four consecutively referred patients to an adult clinical psychology cancer service who met the following inclusion criteria: (i) a total score ≥15 on the Hospital Anxiety and Depression Scale (HADS); (ii) at least 18 years old; (iii) no concurrent psychological treatment; (iv) not actively suicidal; (v) no current substance abuse; (vi) no evidence of psychotic illness; (vii) free from psychotropic medication or on a stable dose for at least 8 weeks; and (viii) sufficient ability in English. All patients were treated as part of the routine service, and this evaluation was approved as a clinical audit at the Royal Liverpool and Broadgreen University NHS Trust (Project reference: AC02660). All patients provided written consent for their data to be used for publication.

### Primary Outcomes

The primary indicator of symptoms was overall level of distress assessed by the HADS-Total (Zigmond and Snaith 1983). The primary indicator of process was the time spent worrying or ruminating, assessed with the cognitive attentional scale-1 (CAS-1; Wells 2009). The results of these two outcome variables are shown in Fig. 2.

#### Hospital Anxiety Depression Scale (HADS; Zigmond and Snaith 1983)

The HADS is a 14-item self-report questionnaire measuring symptoms of anxiety and depression (seven items each) over the past week. Each item is rated on a four-point scale (0–3). Possible scores for each subscale range from 0 to 21, high scores indicating greater anxiety or depression and scores of eight or more indicating casesness. Combining the two subscales provides an overall measure of distress. The HADS-Total is recommended as the optimal outcome measure for evaluating intervention effects on general distress in heterogeneous cancer populations (Luckett et al. 2010).

#### Cognitive Attentional Scale-1 (CAS-1; Wells 2009)

The CAS-1 assesses the core components of the metacognitive model and consists of eight items assessing worry/rumination, threat monitoring and strategies used in response to negative thoughts and metacognitive beliefs. The CAS-1 was designed primarily as a clinical tool to prevent therapist drift when delivering MCT. In the present study, we only used Item 1: “*How much time in the last week have you found yourself dwelling on or worrying about things.”* It is rated on a nine-point scale from 0 (none of the time) to 8 (all of the time).

### Secondary outcomes

#### Fear of Cancer Recurrence Inventory (FCRI; Simard and Savard 2009)

The FCRI is 42-item self-report questionnaire assessing seven aspects of FCR. Each item is rated on a five-point scale (0–4). A total score for FCR is obtained by summing scores on the seven subscales, with higher scores indicating greater severity (range 0–168). The nine-item severity subscale (range 0–36) is used to differentiate clinical from non-clinical FCR. A score of ≥13 is recommended as the optimal cut-off score (Simard and Savard 2015). We report the total FCRI and the FCRI severity subscale to indicate change in overall severity and to show whether participants moved from clinical to non-clinical levels of FCR.

#### Metacognitions Questionnaire-30 (MCQ-30; Wells and Cartwright-Hatton 2004)

The MCQ-30 measures five domains of metacognition by 30 items. Respondents rate how much they “generally agree” with statements presented on a four-point scale from 1 (do not agree) to 4 (agree very much), providing total scores for each subscale ranging from 6 to 24. Higher scores indicate greater conviction in metacognitive beliefs. For this study, we were particularly interested in two subscales; (i) *positive beliefs about worry* (e.g., “Worrying helps me cope”), (ii) *negative beliefs about uncontrollability and danger of worry* (e.g., “When I start worrying I cannot stop”) as these are the key metacognitive beliefs targeted during therapy.

#### Mini International Neuropsychiatric Interview-6.0 (MINI; Sheehan et al. 1998)

The MINI-6.0 is a brief clinical interview to assess psychiatric morbidity and has good reliability and validity for Axis 1 disorders (Lecrubier et al. 1997). It was used to determine if the participants met any current anxiety or depressive disorders according to DSM-IV diagnostic criteria pre-treatment.

#### HADS Anxiety and Depression subscales

We report on whether patients moved from clinical levels of anxiety and depression to non-clinical levels following MCT. to non-clinical cases.

### Procedure

#### Assessment

All patients were assessed by the second author who checked that they met the inclusion criteria and administered the MINI 6.0. During this assessment, which initiated the baseline period, patients completed the HADS, CAS-1, FCRI and MCQ-30. Patients completed the HADS and the CAS-1 weekly to monitor emotional distress and worry/rumination throughout the baseline period. Questionnaires were sent and returned by post. Patients then completed all questionnaires at the end of the baseline in the waiting room immediately before the first treatment session. During the treatment phase, the HADS and the CAS-1 were completed at the beginning of each treatment session. Participants met with the therapist  one week after the end of treatment and at 3- and 6-months follow-up to complete the full set of questionnaires. Patients were also briefly interviewed at the follow-up assessments to identify any significant life events since the end of treatment.

#### Intervention

Treatment followed a manualized protocol (Wells 2009) and session treatment plans and was delivered over a maximum of six individual sessions, each 45–60 min long. Treatment followed the same protocol for patients presenting with different symptoms, including anxiety, depression and fear of cancer recurrence. All therapy was delivered by the second author (AB) who was supervised by the first author (PF) for each patient to ensure adherence to the treatment protocol.

**Fig. 1:**
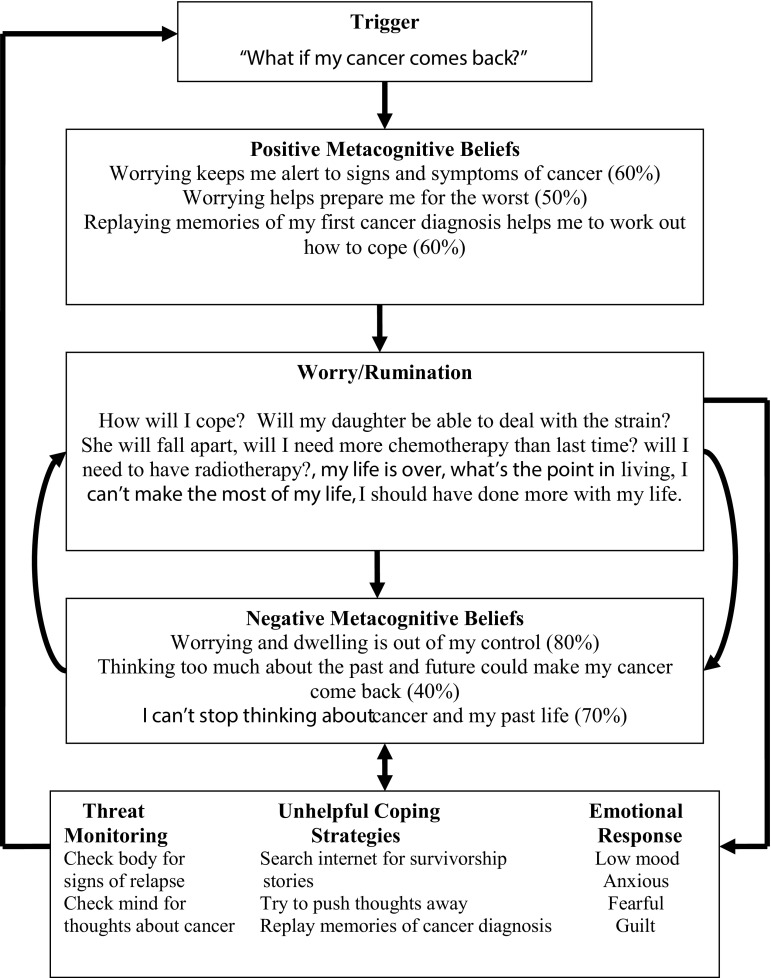
Example of diagrammatic case formulation

In session 1, an idiosyncratic case formulation based on the transdiagnostic metacognitive model (Wells 2009) was developed using the case formulation template (see Fig. 1). Case formulation in MCT involves encapsulating each aspect of the CAS and associated metacognitive beliefs in a manner that clearly explains the maintenance of emotional distress to patients. Socialization to the model followed by sharing the formulation with the patient and using socialisation questions to help the patient understand the impact of worry/rumination and unhelpful coping strategies on distress. The next step was to begin to modify negative beliefs about the uncontrollability of worry/rumination through verbal methods. The therapist initially helped the patients to begin to recognise that worry/rumination is under volitional control by asking questions about the controllability of perseverative thinking e.g. if rumination is completely uncontrollable, how does it ever stop? What happens to your worry, when you are distracted or you need to focus on a specific task? Further modification is achieved by reviewing the evidence and counter evidence that worry/rumination is uncontrollable and training in detached mindfulness (DM) and worry/rumination postponement. DM was first described by Wells and Matthews (1994) and refers to how an individual relates and responds to cognitive events. DM involves enhancing metacognitive awareness and promoting detachment. Metacognitive awareness refers to being aware of one’s cognitive experiences including thoughts, doubts, and memories. Detachment has two aspects; (1) the volitional decision not to engage with or respond to cognitive events with any form of conceptual processing (e.g. worry, rumination, analysing, threat monitoring), (2) the separation of sense of self from the thought, which helps to shift the patient to a metacognitive mode of processing. DM is not a symptom management technique or a form of mediation but instead aims to develop greater flexibility in how a patient responds to thoughts and feeling and enables patients to shift from object mode to a metacognitive mode of processing. DM also helps patients interrupt worry/rumination thereby challenging negative metacognitive beliefs about the uncontrollability of perseverative thinking. Training in DM and worry/rumination postponement starts by enabling patients to differentiate spontaneously occurring negative thoughts and images (e.g. “I’m useless,” “What if my cancer returns?”) from subsequent worry/rumination. Through in-session practice of DM, the therapist enabled patients to develop new ways of responding to negative thoughts, which did not involve worry/rumination, self-focused attention or counterproductive coping strategies. Worry/rumination postponement was presented as a behavioural experiment to challenge the negative metacognitive belief that perseverative thinking is uncontrollable.

In sessions 2 and 3, the therapist primarily focused on reducing negative beliefs about the uncontrollability of worry/rumination and aimed to eliminate any conviction in this belief. In session 4, verbal and behavioural reattribution methods were used to modify positive metacognitive beliefs about the advantages of each aspect of the CAS. This involved reviewing the advantages and disadvantages of worry/rumination, threat monitoring and other coping responses e.g. searching the internet for “positive” survivorship stories. Patients were helped to see that each strategy led to greater worry/rumination, greater distress and an increased conviction in uncontrollability beliefs. If required, behavioural experiments were used to augment belief change achieved by verbal methods. For example, one patient believed that ruination would help her to overcome her low mood by helping her to understand the factors underpinning her depression. A rumination modulation experiment (Wells 2009) was used in which the patient was asked to ruminate in response to negative thoughts on 1 day and to ban rumination on the following day. The patient recognised that rumination made her mood worse, increased apathy levels and provided no solutions, thereby challenging her positive metacognitive beliefs about the usefulness of rumination and resulted in the patient giving up rumination. Sessions 5 and 6 focused on relapse prevention and aimed to reduce any remaining use of the CAS and residual conviction in positive and negative metacognitive beliefs. A therapy ‘blueprint’ was provided for each patient, consisting of a written and diagrammatic case formulation and a treatment summary. A detailed account of the main therapeutic strategies used during treatment was also provided and patients were encouraged to implement these to maintain and further the gains made during treatment.

### Data Analysis

**Fig. 2:**
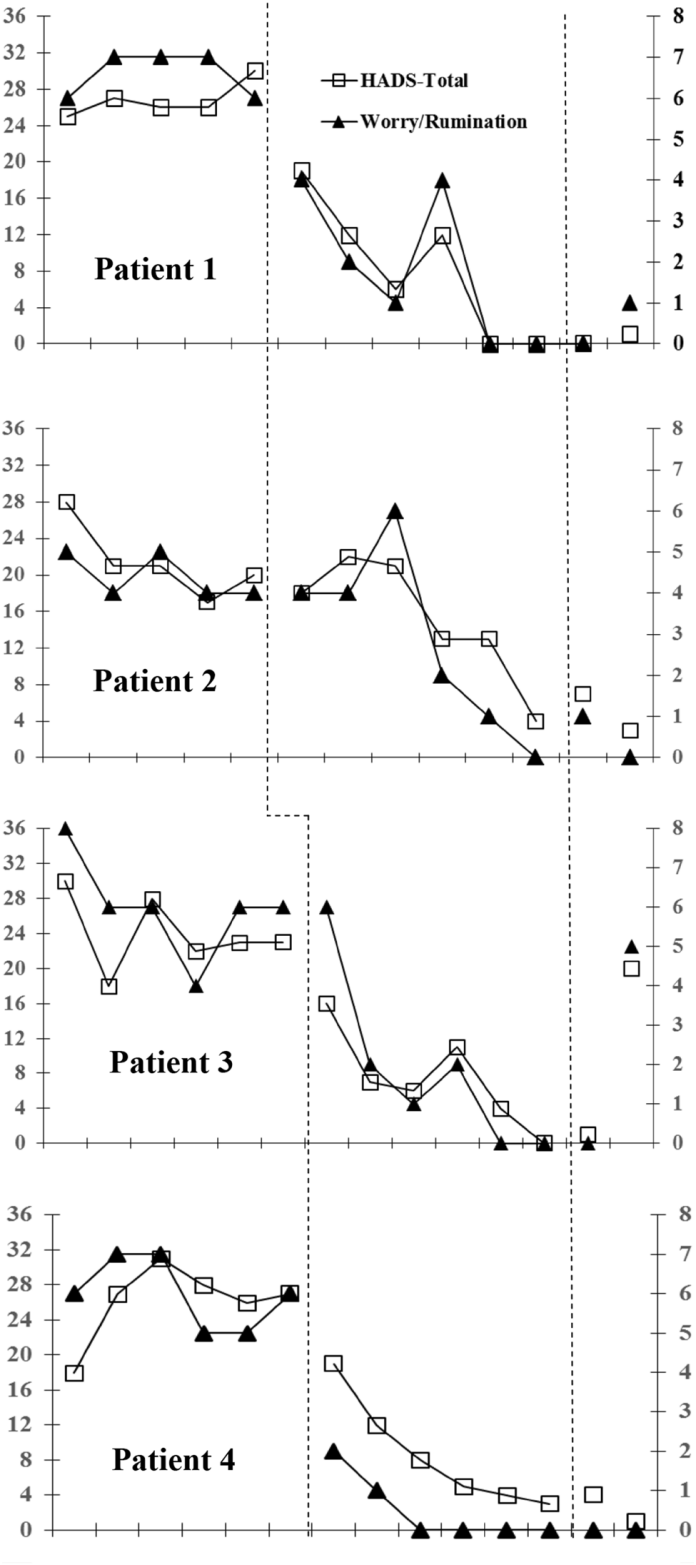
Scores on the Hospital Anxiety and Depression Scale-Total (*left* y-axis) and time spent worrying/ruminating over the previous week (*right* y-axis) across baseline, treatment, and follow-up phases for each patient

**Fig. 3:**
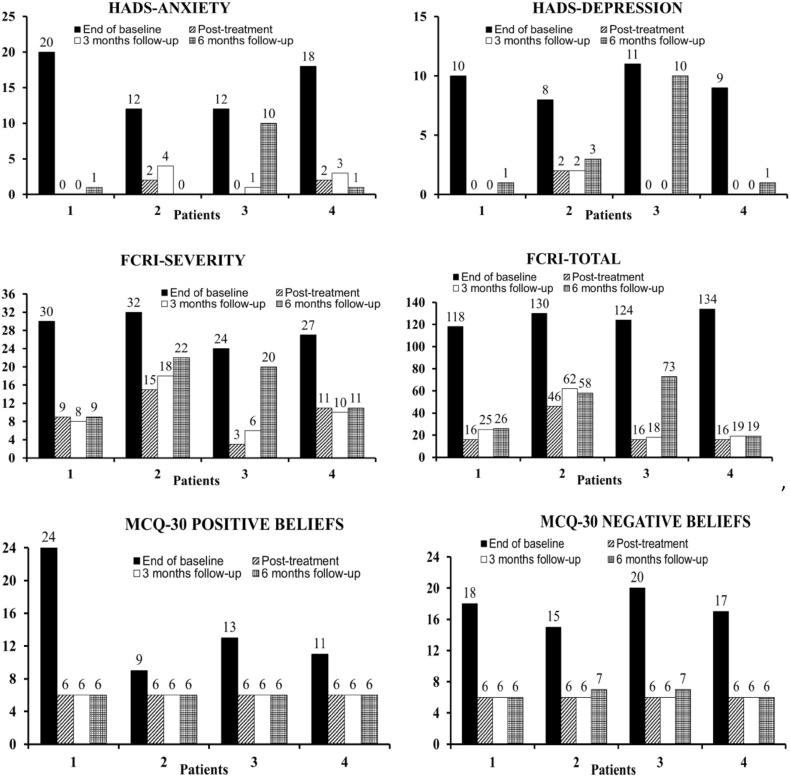
Scores on secondary outcomes scales at end-of-baseline, post-treatment and follow-up for each patient. *FCRI* Fear of Cancer Recurrence Inventory, *HADS* Hospital Anxiety and Depression Scale, *MCQ-30* Metacognitions Questionnaire-30

Evidence of treatment efficacy in single case research is a clear treatment effect following introduction of the intervention. Arguably, visual examination of graphed data provides a stringent test of the effect as only unambiguous effects will be clear (Parsonson and Baer 1992). Therefore, session-by-session scores across baseline, treatment and follow-up on the two primary outcomes (HADS-Total and time spent worrying/ruminating) are graphically illustrated in Fig. 2. For the secondary outcomes, end of baseline, post-treatment and follow-up scores are presented in Fig. 3.

#### Clinically Significant Change

Determining whether change over the course of treatment is clinically significant is a fundamental component of treatment evaluation. The clinical significance of treatment effects on HADS-Total was analyzed using a modified version of the Jacobson method (Jacobson et al. 1999). This allocates each patient to one of four outcomes: reliable deterioration, no change, reliable improvement, or recovered. The first three outcomes are derived from the Reliable Change Index (RCI), which determines whether the change is statistically significant. To be classified as recovered, patients must demonstrate both reliable change and their posttreatment or follow-up scores must be below a cut-off point. It was not possible to use one of the three criteria proposed by Jacobson and colleagues to determine a cut-off point, due to the lack of appropriate normative data for both functional and dysfunctional populations.

Instead, an alternative approach to determining an appropriate cut off point was used. A score of ≥10 on the HADS total provides the optimal threshold when screening for the presence of possible anxiety or depressive disorders (sensitivity 0.80, specificity 0.74; Vodermaier and Millman (2011). Therefore, a cut-off point of ≤9 was chosen to denote recovery. Furthermore, a score of nine on the HADS-Total is equivalent to the mean ($\stackrel{-}{x}$ = 9.82) for an unscreened normative sample (Crawford et al. 2001) and therefore could reasonably be considered to represent a cut-off point below which a HADS total score is more likely to belong to a functional sample. Data to calculate the RCI was drawn from a large sample of cancer patients (Singer et al. 2009) resulting in minimum required change of nine points from pre-treatment to post-treatment or follow-up.

## Results

### Sample Characteristics

Patients’ demographic and clinical characteristics are shown in Table 1. None received any additional psychological or pharmacological treatment for emotional distress during the follow-up period. Participant 1 reduced her antidepressant medication after the 3-month follow-up, under the supervision of her GP. Participant 3 was diagnosed with breast cancer recurrence between the end of treatment and 3-month follow-up and then was investigated for possible lung cancer. Shortly before 6-months follow-up she received an incurable diagnosis. Patient 4 was also investigated for recurrence of breast cancer between the 3- and 6-months follow-ups, but none was found. All patients attended six treatment sessions.

**Table 1:** Participants’ characteristics

	Participant 1	Participant 2	Participant 3	Participant 4
Age (category)	45–55	45–55	45–55	45–55
Gender	Female	Female	Female	Female
Months since cancer diagnosis	26	10	37	28
Months since end of acute medical treatment	20	3	31	27
Cancer diagnosis	Breast cancer	Endometrial cancer	Breast cancer	Breast cancer
Cancer Treatment	Chemotherapy, radiotherapy	Surgery, chemotherapy	Mastectomy, chemotherapy	Mastectomy
Adjuvant cancer treatment	None	None	Tamoxifen	Tamoxifen, zoladex
Relationship Status	Married	Married	Married	Married
Employment Status	Retired	Employed part time	Employed full time	Employed full time
Referrer	General practitioner	Clinical nurse specialist	Clinical oncologist	Clinical oncologist
Previous psychological treatment	CBT	None	CBT, counselling	None
Current psychotropic medication	Citalopram	Citalopram	None	None
Current psychiatric diagnosis	GAD, MDD	None	GAD	GAD

*CBT* cognitive behaviour therapy, *GAD* generalized anxiety disorder, *MDD* major depressive disorder

### Primary Outcomes

Each patient’s score on the HADS-Total and time spent worrying/ruminating during the baseline and treatment phases and at follow-up are shown in Fig. 2. Both outcome measures appeared relatively stable over the baseline, none of the patients had a decreasing or increasing trend in scores over the baseline phase. After treatment began, all patients showed rapid and substantial reductions on the HADS-Total and time spent worrying/ruminating. Furthermore, when the data is examined across phases and patients, scores during the treatment phase are consistently lower than the baseline phase except for the early treatment sessions for patients 2 and 3. Treatment gains were maintained at 3-months follow-up, with three of the four patients maintaining gains at 6-months. The percentage improvement from end-of-baseline to post-treatment on the HADS-Total ranged from 80 to 100%; from the end-of-baseline to 6-months follow-up the range was13–85%.

### Secondary Outcomes

Patients’ end of baseline, post treatment and follow-up scores on HADS-anxiety, HADS-depression, FCRI-Total, FCRI-Severity, and MCQ-30 Positive and Negative beliefs are shown in Fig. 2. Each patient’s post-treatment and 3-months follow-up scores are substantially lower than end-of-baseline scores on all measures. On HADS-anxiety and depression all patients were defined as moderate/severe “cases” at the end of baseline, whereas all scored in the “normal range” at post-treatment and at 3-months follow-up. Except for patient 3, these gains were maintained at 6-months follow-up.

Each patient scored above the cut-off for clinical levels of FCRI-Severity at the end of baseline and had very high scores on the FCRI-Total. At post-treatment and 3-months follow-up, three of the four patients scored in the non-clinical range on FCRI-severity, and two continued to score in the non-clinical range at 6-months’ follow-up. To illustrate the magnitude of reduction in overall FCR, the percentage improvement from end-of-baseline to post-treatment on the FCRI-Total ranged from 64 to 88%; from the end-of-baseline to 6-months follow-up the range was 41–85%. Scores on both positive and negative metacognitive beliefs were also decreased following treatment.

### Clinically Significant Change

At post-treatment and at 3-months follow-up, all patients were recovered, and three patients maintained recovery status at 6-months follow-up. Patient 3 had returned to baseline levels on the HADS and was therefore classified as unchanged at 6-months follow-up.

## Discussion

This study provides initial evidence that MCT delivered over only six sessions can reduce emotional distress in adult survivors of cancer. MCT was associated with large and clinically meaningful improvements in distress, worry and rumination and fear of cancer recurrence. The treatment gains were broadly maintained to 6-months follow, except for the patient who received an incurable diagnosis shortly before that assessment. MCT appeared acceptable and feasible; all patients completed six sessions. Our findings therefore suggest that brief MCT could be an effective intervention to treat psychological morbidity in cancer survivors. Given its transdiagnostic nature, this stands in contrast to the currently influential view that disorder-specific approaches are needed. Although larger studies using a randomized, controlled design are necessary to replicate our findings, and future studies are needed to compare our intervention to other treatments tested to date, It is possible that the S-REF model provides an alternative model on which to base intervention in this population, and may not need to be combined with additional models such as the common-sense model (Leventhal et al. 1992) and relational frame theory (Barnes-Holmes et al. 2001) as proposed in a recent formulation (Fardell et al. 2016).

The case series has limitations. Two participants did not begin treatment at the end of their randomly allocated pre-defined baseline lengths. Instead, one continued her baseline for 2 extra weeks and the other for 1 extra week. This weakened the non-concurrent multiple baseline design as only two different lengths of baseline were used. However, stability was observed over both the 5 and 6-week baseline periods, and outcome measures improved only after treatment began. There was no independent blind assessment of treatment outcome, and all outcomes were assessed by self-report questionnaires which may have led to overestimation of treatment effects. As with all small-N designs, it is not possible to comment on the generalizability of treatment effects to the broader population of cancer survivors. There was no independent rating of adherence to the treatment manual, although treatment adherence was monitored through weekly supervision sessions. In the present study, the outcomes assessed were limited to overall levels of emotional distress, with secondary analyses of anxiety depression and fear of cancer recurrence. Future studies of brief MCT would benefit from assessing a broader array of outcomes including trauma-related symptoms and quality of life.

Overall, the outcomes in this case series suggest that brief MCT has the potential to be a clinically and cost-effective transdiagnostic intervention for adult cancer survivors. In line with the suggested developmental pathway for translating complex interventions proposed by the MRC (2008), the next step is to conduct studies of brief MCT that use larger samples and randomized designs. This would determine whether the approach is efficacious and whether it confers health-economic advantages relative to comparison interventions.
